# Five-Year Outcomes After Fractional Flow Reserve–Guided Deferral of Revascularization in Infarct-Related Artery Lesions

**DOI:** 10.1016/j.jscai.2023.100632

**Published:** 2023-05-02

**Authors:** Hirofumi Ohashi, Shoichi Kuramitsu, Hiroaki Takashima, Hitoshi Matsuo, Kazunori Horie, Hidenobu Terai, Yuetsu Kikuta, Takayuki Ishihara, Tatsuya Saigusa, Tomohiro Sakamoto, Nobuhiro Suematsu, Yasutsugu Shiono, Taku Asano, Kenichi Tsujita, Katsuhiko Masamura, Tatsuki Doijiri, Yohei Sasaki, Manabu Ogita, Tairo Kurita, Akiko Matsuo, Ken Harada, Kenji Yaginuma, Shinjo Sonoda, Tetsuya Amano, Hiroyoshi Yokoi, Nobuhiro Tanaka, J-CONFIRM Investigators

**Affiliations:** a Department of Cardiology, Aichi Medical University, Aichi, Japan; b Department of Cardiology, Kokura Memorial Hospital, Kitakyushu, Japan; c Department of Cardiovascular Medicine, Gifu Heart Center, Gifu, Japan; d Department of Cardiovascular Medicine, Sendai Kousei Hospital, Sendai, Japan; e Department of Cardiology, Kanazawa Cardiovascular Hospital, Kanazawa, Japan; f Department of Cardiology, Fukuyama Cardiovascular Hospital, Fukuyama, Japan; g Kansai Rosai Hospital Cardiovascular Center, Amagasaki, Japan; h Department of Cardiovascular Medicine, Shinshu University School of Medicine, Matsumoto, Japan; i Division of Cardiology, Saiseikai Kumamoto Hospital Cardiovascular Center, Kumamoto, Japan; j Department of Cardiology, Saiseikai Fukuoka General Hospital, Fukuoka, Japan; k Department of Cardiovascular Medicine, Wakayama Medical University, Wakayama, Japan; l Department of Cardiology, St Luke’s International Hospital, Tokyo, Japan; m Department of Cardiovascular Medicine, Graduate School of Medical Sciences, Kumamoto University, Kumamoto, Japan; n Department of Cardiology, Nakamura Hospital, Echizen, Japan; o Department of Cardiology, Yamato Seiwa Hospital, Yamato, Japan; p Department of Cardiology, Chidoribashi Hospital, Fukuoka, Japan; q Department of Cardiology, Juntendo University Shizuoka Hospital, Shizuoka, Japan; r Department of Cardiology and Nephrology, Mie University Graduate School of Medicine, Mie, Japan; s Department of Cardiology, Japanese Red Cross Kyoto Daini Hospital, Kyoto, Japan; t Department of Cardiology, Chubu Rosai Hospital, Nagoya, Japan; u Department of Cardiology, Juntendo University Urayasu Hospital, Chiba, Japan; v Department of Cardiovascular Failure Therapy, Saga University, Saga, Japan; w Department of Cardiology, Fukuoka Sanno Hospital, Fukuoka, Japan; x Department of Cardiology, Tokyo Medical University Hachioji Medical Center, Tokyo, Japan

**Keywords:** coronary artery disease, fractional flow reserve, infarct-related artery

## Abstract

**Background:**

Little evidence is available about the long-term safety of fractional flow reserve (FFR)-guided deferral of revascularization in infarct-related artery (IRA) lesions, especially when measuring FFR in the late setting after myocardial infarction (MI). This study aimed to assess the long-term outcomes after deferral of revascularization in IRA lesions based on FFR assessed in the late phase of post-MI.

**Methods:**

From the J-CONFIRM registry (Long-Term Outcomes of Japanese Patients With Deferral of Coronary Intervention Based on Fractional Flow Reserve in Multicenter Registry), data on 1447 lesions (1263 patients) were divided into 2 groups: the IRA and non-IRA groups. The primary study end point was the cumulative 5-year incidence of target vessel failure (TVF), such as cardiac death, target vessel–related MI, and clinically driven target vessel revascularization.

**Results:**

Of the 1447 lesions, 138 (9.5%) were classified into the IRA group. The median duration of FFR measurement was 716 days after MI. The frequency of visual-functional mismatches (ie, FFR >0.80 and percent diameter stenosis ≥50% or FFR ≤0.80 and percent diameter stenosis <50%) was comparable between the IRA and non-IRA groups (31.9% vs 36.3%). The cumulative 5-year incidence of TVF did not differ between the groups (9.2% vs 11.8%; inverse probability–weighted hazard ratio, 1.18, 95% confidence intervals, 0.48-2.91, *P* = .71). Similar results were observed irrespective of regional wall motion assessed by ultrasonic cardiography and acute MI type.

**Conclusions:**

The 5-year TVF rate did not differ between the IRA and non-IRA lesions when deferring revascularization guided by FFR in the late setting of post-MI.

## Highlights

• Of 1447 lesions, 138 (9.5%) were classified into the infarct-related artery group.
• Visual-functional mismatches were similarly observed in the two groups.
• Five-year target vessel failure did not differ between the two groups.

## Central Illustration

**Figure:**
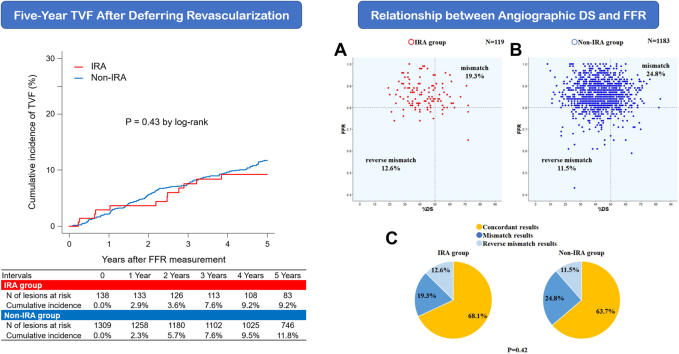

## Introduction

The diagnostic and prognostic significance of fractional flow reserve (FFR) has been well established in coronary artery stenosis supplying the normal myocardial region.^1^ Previous studies showed that the basal and hyperemic coronary flow decreased in infarcted myocardial regions, mainly owing to the reduced oxygen consumption and inadequate dilation of epicardial and microvascular arterioles,^2^^,^^3^ raising concerns about FFR measurement in infarct-related artery (IRA) lesions. However, De Bruyne et al^4^ validated the diagnostic accuracy of FFR in identifying the functional significance of IRA lesions. Furthermore, Beleslin et al^5^ reported that the FFR assessment before revascularization might predict the improvement in cardiac function of patients with previous myocardial infarction (MI). Although these findings supported the diagnostic validity of FFR measurement in patients with previous MI, these lesions were not revascularized at the timing of the index MI. The extent of microvascular damage after revascularizing IRA lesions differs between individuals, potentially affecting FFR values and future adverse outcomes in IRA lesions.^6^^,^^7^ However, little evidence is available regarding the long-term outcomes after deferral of revascularization based on FFR in IRA lesions revascularized at the index MI, especially when measuring FFR in the late phase after MI. This study aimed to assess the 5-year clinical outcomes of FFR-guided deferral of revascularization in the IRA lesions compared with those in non-IRA lesions by analyzing a large-scale registry.

## Methods

### Study population

This study is a post hoc analysis of the J-CONFIRM registry (Long-Term Outcomes of Japanese Patients With Deferral of Coronary Intervention Based on Fractional Flow Reserve in Multicenter Registry) designed to investigate the clinical outcomes of Japanese patients with deferral of revascularization based on FFR measurement in real-world practice. The study design and 2-year and 5-year results have been reported previously.^8^^,^^9^ In brief, this registry included 1263 patients with 1447 angiographically intermediate coronary artery lesions in whom revascularization was deferred based on FFR measurement between September 2013, and June 2015. Patients with (1) acute MI, (2) cardiogenic shock, (3) a chronic total occlusion lesion, (4) a graft lesion, or (5) limited life expectancy owing to comorbidity were excluded from this registry.

For this, we classified the lesions into the IRA and non-IRA groups. The IRA was defined as a coronary artery lesion responsible for an acute myocardial infarction (AMI), including ST-segment elevation myocardial infarction (STEMI) and non–ST-segment elevation myocardial infarction (NSTEMI). IRA was identified by each site investigator mainly based on coronary angiographic findings, in addition to various examination results (eg, electrocardiogram, ultrasonic cardiography, and intravascular imaging devices).

### Study end points and definition

The primary study end point was the cumulative 5-year incidence of target vessel failure (TVF), such as cardiac death, target vessel–related myocardial infarction (TVMI), and clinically driven target vessel revascularization (CD-TVR). Clinically driven target lesion revascularization (CD-TLR) was also assessed. Cardiac death, TVMI, CD-TVR, and CD-TLR were defined according to the Academic Research Consortium definition as previously described.^8^, ^9^, ^10^ The secondary study end points included the following: (1) the relationship between angiographic diameter stenosis (DS) and FFR in the IRA and non-IRA groups and (2) the cumulative 5-year incidence of TVF in the IRA group according to the AMI type (STEMI or NSTEMI) and regional wall motion abnormalities subtended by IRA lesions. At the baseline procedure, the regional wall motion was assessed by ultrasonic cardiography and was divided into 2 groups: normal or abnormal (hypokinetic or akinetic).^11^

### Data collection and clinical follow-up

All baseline and clinical follow-up data were prospectively collected using a dedicated electronic case report form. Site investigators at each institution obtained them at outpatient clinic visits or by telephone contacts with patients, relatives, or referring physicians at 12, 24, 36, 48, and 60 months after the index procedure. All clinical events were adjudicated by an independent clinical events committee.

### Statistical analysis

Categorical variables are expressed as number and percentages. Continuous variables are indicated as mean ± standard deviation or median (lower and upper quartiles). Correlation between FFR and %DS was analyzed using the Pearson correlation coefficient. The cumulative incidence rates of study end points after FFR measurements were estimated using the Kaplan-Meier method and compared using the log-rank test. Hazards for TVF, cardiac death, CD-TLR, CD-TVR, and TVMI were compared between the IRA and non-IRA groups on a patient or lesion basis, by fitting the Cox models and adjusting for clinically determined possible risk factors (listed in Tables 1 and 2). For a lesion-level analysis, the robust sandwich variance estimators aggregating the score residuals at the patient level were used. To confirm the robustness of the results, we also performed an inverse probability–weighted Cox analysis with the robust variance estimators, followed by the estimation of propensity scores for IRA conditional on the same covariates using logistic models.

**Table 1:** Baseline clinical characteristics of the study groups.

	IRA	Non-IRA	*P*
No. of patients	128	1135	
Age, y^a^	69.5 (62.0-75.0)	71.0 (65.0-77.0)	.053
Male sex^a^	102 (79.7)	838 (73.8)	.17
Hypertension^a^	100 (78.1)	869 (76.6)	.74
Diabetes mellitus^a^	53 (41.4)	426 (37.5)	.39
Dyslipidemia^a^	97 (75.8)	712 (62.7)	.003
Current smoking^a^	43 (33.6)	360 (31.7)	.69
Hemodialysis^a^	3 (2.3)	62 (5.5)	.20
Prior myocardial infarction^a^	128 (100)	237 (20.5)	<.001
Previous PCI^a^	120 (93.8)	628 (55.3)	<.001
Previous CABG^a^	6 (4.7)	26 (2.3)	.13
LVEF, %	59.0 (50.9-69.3)	64.6 (57.0-70.0)	.001
≤40%^a^	9 (8.4)	43 (4.5)	.09
Clinical symptom			.65
Asymptomatic	69 (53.9)	580 (51.1)	
CCS I	42 (32.8)	412 (36.3)	
CCS II	11 (8.6)	107 (9.4)	
CCS III	4 (3.1)	18 (1.6)	
CCS IV	2 (1.6)	18 (1.6)	
AMI type			
STEMI	68 (49.3)	–	
NSTEMI	32 (23.2)	–	
Unknown	38 (27.5)	–	
Medication at discharge			
Antiplatelet therapy^a^	125 (97.7)	947 (83.4)	<.001
β-Blocker^a^	65 (50.8)	356 (31.4)	<.001
Ca channel blocker^a^	61 (47.7)	595 (52.4)	.35
ACEI/ARB^a^	83 (64.8)	650 (57.3)	.11
Nitrate^a^	33 (25.8)	208 (18.3)	.06
Statin^a^	100 (78.1)	716 (63.1)	.001
Oral hypoglycemic agents^a^	40 (31.3)	293 (25.8)	.20
Insulin^a^	1 (0.8)	55 (4.8)	.04

Categorical variables are expressed as number (%) and continuous variables as median (interquartile range).

ACEI, angiotensin-converting enzyme inhibitor; ARB, angiotensin receptor blocker; CCS, Canadian Cardiovascular Society; CABG, coronary artery bypass grafting; FFR, fractional flow reserve; IRA, infarct-related artery; LVEF, left ventricular ejection fraction; NSTEMI, non–ST-segment elevation myocardial infarction; PCI, percutaneous coronary intervention; STEMI, ST-segment elevation myocardial infarction.

a Variables adjusted for in multivariable Cox models and inverse probability–weighted Cox models.

**Table 2:** Lesion characteristics and FFR measurements

	IRA	Non-IRA	*P*
No. of lesions	138	1309	
Target vessel			.44
Left main coronary artery^a^	5 (3.6)	32 (2.4)	
Left anterior descending coronary artery^a^	68 (49.3)	635 (48.5)	
Left circumflex coronary artery^a^	25 (18.1)	302 (23.1)	
Right coronary artery^a^	40 (29.0)	345 (26.4)	
ACC/AHA lesion classification			.39
A	13 (9.4)	150 (11.5)	
B1	48 (34.8)	362 (27.7)	
B2	53 (38.4)	543 (41.6)	
C	24 (17.4)	251 (19.2)	
Mean FFR^a^	0.85 (0.81-0.89)	0.86 (0.82-0.90)	.43
FFR category			.44
<0.75	3 (2.2)	50 (3.8)	
0.75-0.80	22 (15.9)	158 (12.1)	
0.81-0.90	84 (60.9)	787 (60.1)	
0.91-1.00	29 (21.0)	314 (24.0)	
Angiographic findings			
Bifurcation lesion^a^	42 (33.9)	367 (30.6)	.48
Tortuous lesion^a^	20 (16.1)	226 (18.8)	.54
Calcified lesion^a^	21 (16.9)	164 (13.7)	.32
In-stent restenosis lesion^a^	33 (23.9)	72 (5.5)	<.001
Quantitative coronary analytic results			
Reference vessel diameter, mm	2.67 (2.27-3.16)	2.76 (2.36-3.22)	.45
≤2.5 mm^a^	43 (36.1)	401 (33.9)	.61
Minimum lumen diameter, mm	1.54 (1.29-1.85)	1.56 (1.29-1.85)	.73
Diameter stenosis, %	43.0 (33.8-50.1)	0 43 (36.8-51.0)	.76
≥50%^ax^	30 (25.2)	350 (29.6)	.34
Lesion length, mm	11.7 (9.1-14.4)	11.6 (9.3-15.3)	.78
≥20.0 mm^a^	11 (8.9)	108 (9.0)	1.00

Categorical variables are expressed as number (%) and continuous variables as median (interquartile range).

ACC/AHA, American College of Cardiology/American Heart Association; FFR, fractional flow reserve; IRA, infarct-related artery.

a Variables adjusted for in multivariable Cox models and inverse probability–weighted Cox models.

All statistical analyses were performed by 2 physicians (H.O., S.K.) using the R software (version 3.5.2; R Foundation for Statistical Computing). A 2-sided *P* value of <.05 was considered statistically significant.

## Results

### Study population

Of the 1447 lesions, 138 (9.5%) lesions were classified into the IRA group (Supplemental Figure S1). The 5-year clinical follow-up was completed in 92.0% and 94.5% of the patients in the IRA and non-IRA groups, respectively.

### Baseline clinical characteristics

The baseline clinical characteristics are provided in Table 1. Most patients presented with chronic coronary syndrome (CCS). Compared with the non-IRA group, the IRA group had a higher prevalence of dyslipidemia, previous MI, previous percutaneous coronary intervention (PCI), previous coronary artery graft bypass, and a lower left ventricular ejection fraction. The prevalence of antiplatelet therapy, β-blocker, nitrate, statin, and insulin use at discharge was significantly higher in the IRA group than that in the non-IRA group. Regarding lesion characteristics, no significant differences were observed between the 2 groups, except for in-stent restenosis lesions (Table 2). Supplemental Table S1 summarizes the detailed information on IRA lesions. Revascularizing IRA lesions at the index AMI events was performed in 85.5% (118/138) of the patients. The location of all IRA lesions was identified primarily owing to severe coronary stenosis in patients with NSTEMI. Among them, 90.6% (29/32) underwent revascularization; the remaining were treated medically owing to the proven epicardial vasospastic angina (n = 2) or relatively small myocardial territory (n = 1).

### FFR measurement

The median duration from AMI occurrence to FFR measurement was 716 days (255-3008 days). The median FFR did not differ between the IRA and non-IRA groups (0.85 [0.81, 0.89] vs 0.86 [0.82, 0.90]; *P* = 0.43), regardless whether %DS of ≥50% or not (Table 2 and Supplemental Figure S2). Most lesions showed FFR of >0.80, whereas those with FFR ≤0.80 were observed in 18.1% and 15.9% of the IRA and non-IRA groups, respectively (Table 2).

### Relationship between angiographic DS and FFR

There was a weak correlation between the FFR and %DS values in the IRA group (correlation coefficient, −0.18, 95% confidence interval [CI], −0.135 to −0.003; *P* = .046) (Central Illustration), whereas no correlation was observed in the non-IRA group (correlation coefficient, 0.014; 95% CI, −0.043 to 0.071; *P* = .64) (Central Illustration). In the IRA group, 23 lesions (19.3%) showed FFR of >0.80 and %DS of ≥50 (a mismatch), whereas 15 lesions (12.6%) showed FFR of ≤0.80 and %DS of <50 (a reverse mismatch) (Central Illustration). In the non-IRA group, mismatch and reverse mismatch were observed in 293 lesions (24.8%) and 136 lesions (11.5%), respectively (Central Illustration). The difference and ratio between overall mismatch rates in the IRA (38/119, 31.9%) and non-IRA (429/1183, 35.9%) groups were −4.3% (95% CI, −13.1% to 4.5%) and 0.88 (95% CI, 0.67 to 1.16), respectively (Central Illustration).

**Central Illustration:**
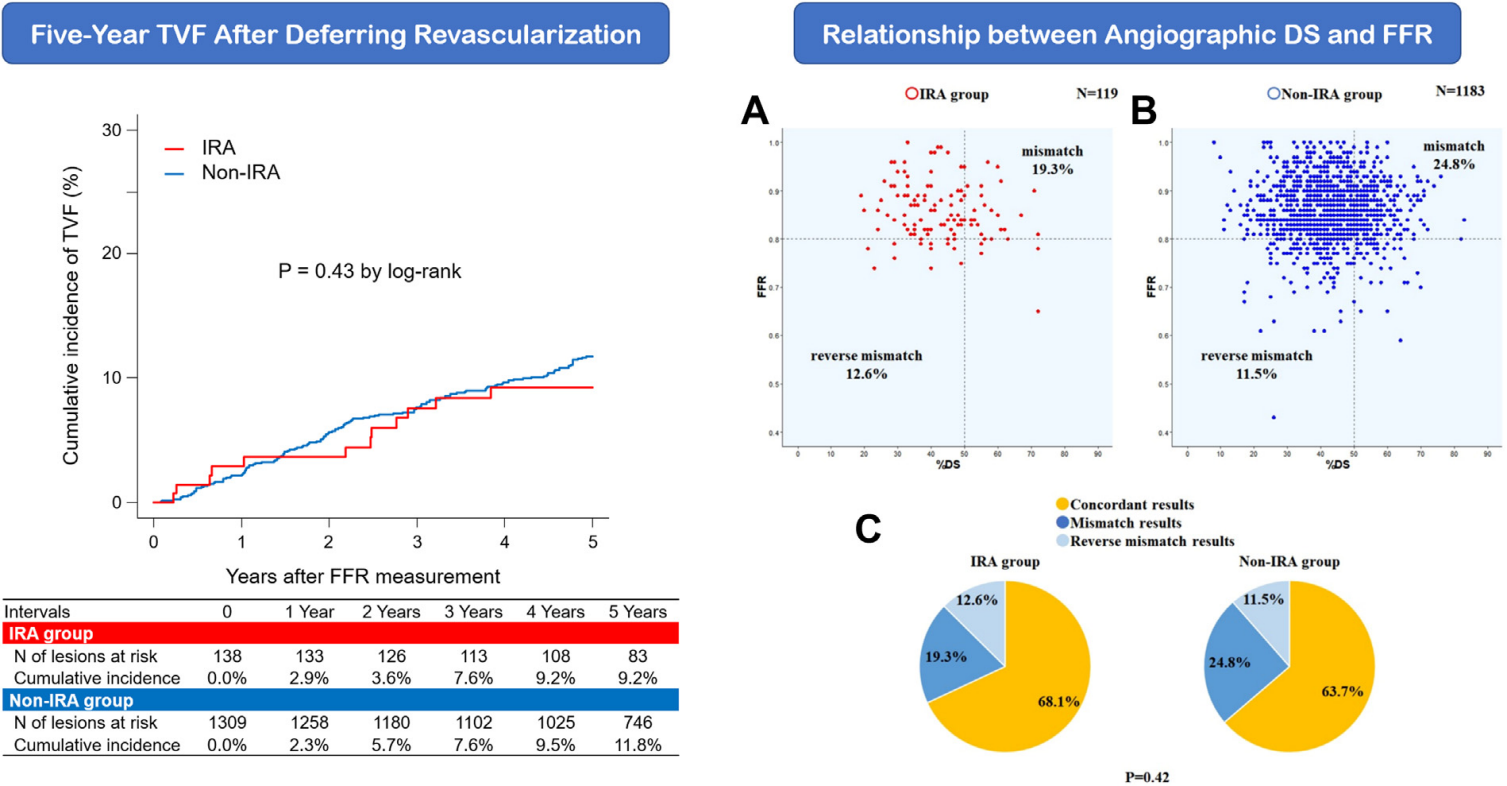
**Five-year TVF after deferring revascularization based on fractional flow reserve (FFR) and the relationship between angiographic diameter stenosis (DS) and FFR.** (A) IRA group, (B) non-IRA group, and (C) the frequency of discordance between angiographic DS and FFR in the IRA and non-IRA groups. Data on percentage diameter stenosis were not available in 145 lesions (IRA group, n = 19; non-IRA group, n = 126). IRA, infarct-related artery; PCI, percutaneous coronary intervention; TVF, target vessel failure.

### Clinical outcomes

**Figure 1:**
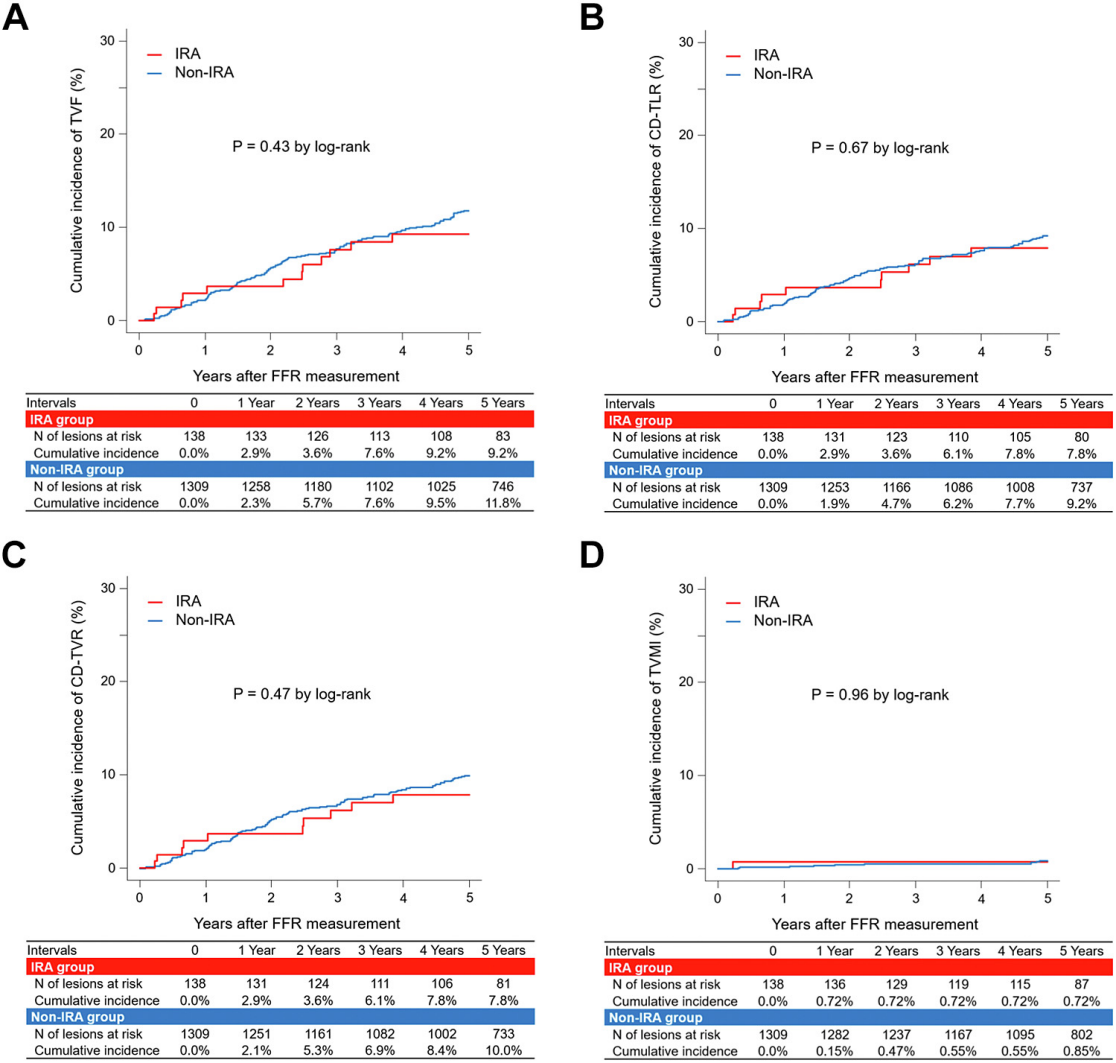
**Clinical events after deferral of revascularization through 5 years on a lesion basis.** (A) Target vessel failure (TVF), (B) clinically driven target lesion revascularization (CD-TLR), (C) clinically driven target vessel revascularization (CD-TVR), and (D) target vessel–related myocardial infarction (TVMI). IRA, infarct-related artery; FFR, fractional flow reserve.

The cumulative 5-year incidence of TVF did not differ between the IRA and the non-IRA groups (9.2% vs 11.8%, inverse probability–weighted hazard ratio [HR], 1.18; 95% CI, 0.48-2.91; *P* = .71) (Figure 1, Central Illustration and Supplemental Table S2). Similarly, the cumulative 5-year incidences of CD-TLR, CD-TVR, and TVMI were comparable between the 2 groups (7.8% vs 9.2%, inverse probability–weighted HR, 1.31; 95% CI, 0.45 to 3.80; *P* = .62; 7.8% vs 10.0%, inverse probability–weighted HR, 1.13; 95% CI, 0.41-3.12; *P* = .81; and 0.72% vs 0.85%, inverse probability–weighted HR, 0.47; 95% CI, 0.06-3.80, *P* = .48, respectively) (Figure 1 and Supplemental Table S2). Cardiac death rarely occurred in both groups during the 5-year follow-up (1.7% vs 2.0%; inverse probability–weighted HR, 0.61; 95% CI, 0.08-4.34; *P* = .62) (Supplemental Figure S3 and Supplemental Table S3).

### The effect of wall motion abnormalities and AMI type on FFR measurement and outcomes

The frequency of visual-functional mismatches was comparable irrespective of the AMI type and regional wall motion abnormalities (Supplemental Figures S4 and S5**)**. Regarding outcomes, the cumulative 5-year incidence of TVF did not significantly differ between the abnormal and normal groups (10.4% vs 10.3%; *P* = .93) (Supplemental Figure S6A). Consistently, no significant difference in the 5-year TVF was observed between the STEMI and NSTEMI groups (10.8% vs 11.9%; *P* = 0.79) (Supplemental Figure S6B).

## Discussion

The main findings of this study are as follows: (1) the frequency of visual-functional mismatches between angiographical DS and FFR was comparable between the IRA and non-IRA groups when measuring FFR in the late phase after MI; (2) the cumulative 5-year incidence of TVF did not differ between the IRA and non-IRA groups; and (3) the regional wall motion abnormalities and AMI type did not affect the FFR results and 5-year outcomes after FFR-guided deferral of revascularization.

### Frequency of visual-functional mismatch in IRA lesions

Coronary angiography often underestimates or overestimates a lesion’s functional severity.^12^ Previous studies demonstrated that the discordance between angiographic DS and FFR (ie, visual-functional mismatches) was ≈30% to 40% of lesions.^13^ From a pathophysiologic viewpoint, IRA lesions have 2 different features from non-IRA lesions: (1) a smaller myocardium mass for the same degree of coronary stenosis and (2) dysfunction of microcirculation.^12^ However, the diagnostic accuracy of FFR in IRA lesions has been validated because the concept of FFR in IRA lesions considers both coronary stenosis and its myocardial perfusion area.^4^ Conversely, to date, little data are available regarding the relationship between the angiographic DS and FFR in IRA lesions, especially when FFR was measured in the late phase after MI. In this, the frequency of visual-functional mismatches in IRA and non-IRA lesions was 31.9% and 35.9%, respectively; the clinically relevant difference in mismatch rates between the 2 groups seemed implausible. In addition, FFR values were similar between the IRA and non-IRA lesions, irrespective of whether the %DS was ≥50% or not. Recently, Cuculi et al^6^ reported that hyperemic coronary flow significantly increased with the decreasing index of microcirculatory resistance at the 6-month follow-up after STEMI, suggesting that coronary microcirculation generally recovers after STEMI in IRA lesions over time. These temporal changes in coronary physiology in IRA lesions might help understand the comparable frequency of visual-functional mismatch between the 2 groups in the late phase after MI, although the microvascular function could not be assessed in this. Accordingly, this study suggested the validity of FFR measurement for identifying the functional significance of IRA lesions in the late phase after MI.

### Long-term outcomes after deferring revascularization in IRA lesions

Physiologic lesion severity has been closely related to plaque vulnerability, and both components affect future cardiovascular events.^14^^,^^15^ Intriguingly, Lee et al^15^ reported that coronary lesions with high-risk plaque characteristics increased the risk of vessel-oriented composite outcomes than those without high-risk plaque characteristics when FFR showed >0.80, whereas the relationship was not observed in those with FFR ≤ 0.80. Previous studies demonstrated that patients with acute coronary syndrome (ACS) feature pan-coronary plaque vulnerability and advanced atherosclerosis; major adverse cardiovascular events during the follow-up were attributable to recurrences at the site of culprit lesions and to nonculprit lesions in patients with ACS.^16^^,^^17^ Because the plaque vulnerability may affect adversely on the outcomes even when FFR showed >0.80, some concerns have been raised regarding the safety of FFR-guided deferral of revascularization in IRA lesions. Indeed, previous studies reported that vulnerable plaque increased the risk of future cardiovascular events even when FFR is >0.80.^15^^,^^18^ Moreover, Hakeem et al^19^ reported that deferring revascularization based on FFR of ≥0.75 was associated with worse outcomes in patients with ACS than that in those with CCS. This study demonstrated that the cumulative 5-year incidence of TVF did not differ between the IRA and non-IRA groups, even after adjustment for baseline characteristics, which was not in line with the previous study.^19^ Possible explanations for this were as follows: (1) the timing of FFR measurement differed between the present and previous studies (chronic vs acute phase after MI); and (2) most patients underwent revascularization for IRA lesions in this study, in contrast to that observed in the previous study. To our best knowledge, this is the first to assess the long-term outcomes of FFR-guided deferral of revascularization in IRA lesions (mostly revascularized at the index AMI events) in the late phase after MI. Our results suggested that IRA lesions could be safely deferred based on FFR and non-IRA lesions in a chronic setting. However, because this could not assess how many vulnerable plaques were included in IRA lesions, further studies are mandatory to validate our results.

### Effect of wall motion and AMI type on FFR assessment

Previous studies demonstrated that FFR could accurately identify inducible ischemia on myocardial perfection scintigraphy in IRA lesions.^4^^,^^20^ However, given that these studies measured FFR in nonocclusive IRA lesions early after AMI (ie, 2-30 days), it remains unclear whether previous results could apply to patients with occlusive IRA lesions who had undergone primary PCI at the timing of AMI occurrence. This study demonstrated the long-term safety of FFR-guided deferral of revascularization in such patients in the chronic setting, thereby filling a gap between previous studies and daily practice. On the contrary, myocardial damage after AMI varies widely between individuals, being affected by several aspects (eg, the timing of reperfusion, clinical presentation, lesion location, or microvascular damage).^21^^,^^22^ In this study, the frequency of visual-functional mismatches seemed not different regardless of baseline clinical presentation (STEMI or NSTEMI) and wall motion abnormalities at the follow-up. Similarly, the cumulative 5-year incidence of TVF was not different in each group. These findings suggested that coronary microcirculation mostly recovered when measuring FFR in the late phase after MI, underscoring the reliability of FFR assessment in various conditions of IRA lesions. By contrast, these results might be hampered by several limitations of this study (eg, no data on coronary microvascular function and maximal creatine kinase, various timing of FFR measurement, and a relatively small number of the IRA group). Further investigations should address the diagnostic and prognostic significance of FFR in IRA lesions.

### Clinical implications

Primary PCI can restore epicardial coronary blood flow in patients with AMI, dramatically reducing their mortality rates.^22^ However, despite complete revascularization, microvascular damage exists in ≈50% of cases, limiting FFR use in an acute setting.^7^ By contrast, coronary microcirculation generally recovers over time in patients with AMI undergoing primary PCI, so FFR could theoretically be used to identify the functional significance of IRA lesions in the chronic phase.^7^ Notably, the extent of microvascular damage differs between individuals, potentially affecting FFR values and future adverse outcomes.^6^ In addition, recent studies showed that previous MI was independently associated with a higher risk of cardiovascular death in patients with CCS; recurrent events frequently occurred in culprit lesions even after revascularization in patients with ACS.^16^^,^^23^ Although these findings concern whether the feasibility and safety of FFR in IRA lesions would be as assured as in non-IRA lesions, no evidence exists to date. This study is the first to evaluate the frequency of visual-functional mismatch and long-term outcomes after deferring revascularization in IRA lesions (mostly revascularized at the timing of the index MI) when measuring FFR in the late phase after MI. Our results suggest that FFR can be used in daily practice for deferring revascularization in IRA lesions and non-IRA lesions in patients with CCS when measuring FFR in the late setting of post-MI.

### Limitations

There are several limitations to this study. First, this study was a post hoc analysis of the J-CONFIRM registry; therefore, the sample size could not be calculated. Because patients with ACS generally experience a higher risk profile than those with CCS, physicians might be more likely to adhere to optimal medical therapy in patients with ACS. Indeed, the IRA group took anti-ischemic drugs (eg, antiplatelet therapy, β-blockers, and statins) more frequently than that by the non-IRA group in this study. Although we performed multivariable and inverse probability–weighted Cox methods to adjust for the differences in baseline characteristics between the groups, unmeasured confounding factors might have biased this study results. Second, identifying IRA in cases with NSTEMI is challenging because patients are more likely to present with multivessel or nonobstructive coronary artery disease.^24^ This study determined all IRA lesions in patients with NSTEMI primarily due to angiographically severe coronary stenosis. However, the diagnostic accuracy of coronary angiography for identifying IRA has been limited.^24^ Furthermore, the AMI type was unknown in 38 of the 138 (27.5%) patients with IRA, although 22 of 38 (57.9%) underwent revascularization at the MI events. These findings might have biased the conclusions in this study. Third, cardiac magnetic resonance and cardiac perfusion images are recommended to assess the myocardial viability of the infarcted area.^25^ However, these modalities were not available in this study. Moreover, we could not obtain any information on detailed ultrasonic cardiography findings except for wall motion abnormalities. These findings might have affected the conclusions of this study. Fourth, measuring coronary flow reserve and index of microcirculatory resistance would be informative to understand how coronary microvascular function in IRA lesions recovered at FFR measurement, whereas this study did not owing to the retrospective study design. Further studies are required to investigate the effect of coronary microvascular function on FFR assessment in IRA lesions in the late phase after MI. Finally, although we speculated that IRA lesions might lead to worse outcomes than non-IRA lesions, this should be considered hypothesis generating owing to no comparison of results between IRA and non-IRA lesions based on FFR-guided decision making (revascularization vs medical therapy) and different timing of FFR measurement (early vs late).

## Conclusions

When deferring revascularization based on FFR in the late setting of post-MI, the 5-year clinical outcomes did not differ between the IRA and non-IRA lesions. Our results suggested the long-term safety of FFR-guided deferral of revascularization in IRA lesions in clinical practice, whereas further studies are warranted to elucidate our hypothesis.
