# Supplementary material for: Five-Year Outcomes After Fractional Flow Reserve–Guided Deferral of Revascularization in Infarct-Related Artery Lesions
Source: J Soc Cardiovasc Angiogr Interv. 2023 May 2;2(3):100632. doi: 10.1016/j.jscai.2023.100632 (PMC11307784; doi:10.1016/j.jscai.2023.100632)
Supplement: Supplementary Material [file mmc1.docx]

**SUPPLEMENTARY MATERIALS**

**Appendix: List of participating centers and the investigators**

Aichi Medical University: Hiroaki Takashima, Hirofumi Ohashi

Chidoribashi Hospital: Fumitoshi Toyota, Yohei Sasaki

Chubu Rosai Hospital: Ken Harada

Fukuoka Sanno Hospital: Hiroyoshi Yokoi, Yasunori Yamamoto

Fukuyama Cardiovascular Hospital: Yuetsu Kikuta, Mika Hirabayashi

Gifu Heart Center: Hitoshi Matsuo, Jun Kikuchi, Ryosuke Itakura

Japanese Red Cross Kyoto Daini Hospital: Akiko Matsuo

Juntendo University Shizuoka Hospital: Satoru Suwa, Manabu Ogita

Juntendo University Urayasu Hospital: Ken Yokoyama, Kenji Yaginuma, Hiroyuki Isogai

Kainan Hospital: Takashi Yamada, Noriyoshi Kanemura

Kanazawa Cardiovascular Hospital: Hidenobu Terai

Kansai Rosai Hospital Cardiovascular Center: Takayuki Ishihara

Kumamoto University: Kenichi Tsujita, Kenshi Yamanaga, Masahiro Ishii, Michiyo Saito

Kokura Memorial Hospital: Shoichi Kuramitsu

Mie University Graduate School of Medicine: Tairo Kurita

Nakamura Hospital: Katsuhiko Masamura, Masanori Kanehachi

Narita Memorial Hospital: Toru Niwa

Nayoro City General Hospital: Masaru Yamaki

Saiseikai Fukuoka General Hospital: Nobuhiro Suematsu

Saiseikai Kumamoto Hospital: Tomohiro Sakamoto, Kazuhisa Kodama

Sendai Kosei Hospital: Kazunori Horie

Shinshu University: Tatsuya Saigusa, Yasushi Ueki, Keisuke Senda

St Luke’s International Hospital: Atsushi Mizuno, Taku Asano

Tajimi City Hospital: Daiki Kato

Tokyo Medical University: Nobuhiro Tanaka, Jun Yamashita

University of Occupational and Environmental Health Japan School of Medicine: Shinjo Sonoda, Reo Anai

Wakayama Medical University: Yasutsugu Shiono

Yamato Seiwa Hospital: Tatsuki Doijiri

**Supplementary Table 1: Detailed Information on Infarct-Related Artery**

| **Variables** | **IRA (n=138)** |
| --- | --- |
| Treatment at the index AMI events |  |
| PCI | 114 (82.6%) |
| CABG | 4 (2.9%) |
| Medical therapy | 7 (5.1%) |
| Unknown | 13 (9.4%) |
| Wall motion at OMI site* |  |
| Normal | 42 (30.4%) |
| Hypokinesis | 45 (32.6%) |
| Akinesis | 35 (25.4%) |
| Unknown | 16 (11.6%) |

Data are presented as number (percentage). *Assessed by ultrasonic cardiography at the time of fractional flow reserve measurement.

AMI = acute myocardial infarction; CABG = coronary artery bypass graft; IRA = infarct-related artery; NSTEMI = non-ST-segment elevation myocardial infarction; OMI = old myocardial infarction; PCI = percutaneous coronary intervention; STEMI = ST-segment elevation myocardial infarction

**Supplementary Table 2:** **Adjusted Risk of Clinical Events Through 5 Years on a Lesion Basis**

|  | **Cumulative 5-year incidence** | |  | **Crude HR** | |  | **Multivariable adjustment**† | |  | **IPW adjustment**† | |
| --- | --- | --- | --- | --- | --- | --- | --- | --- | --- | --- | --- |
| **Outcome** | **IRA** | **Non-IRA** |  | **HR (95% CI)*** | ***p**** |  | **HR (95% CI)*** | ***p**** |  | **HR (95% CI)*** | ***p**** |
| TVF | 9.2% | 11.8% |  | 0.78 (0.43-1.42) | 0.43 |  | 1.33 (0.61-2.92) | 0.47 |  | 1.18 (0.48-2.91) | 0.71 |
| CDTLR | 7.8% | 9.2% |  | 0.87 (0.45-1.67) | 0.67 |  | 1.65 (0.68-3.99) | 0.26 |  | 1.31 (0.45-3.80) | 0.62 |
| CDTVR | 7.8% | 10.0% |  | 0.79 (0.41-1.51) | 0.47 |  | 1.36 (0.57-3.21) | 0.49 |  | 1.13 (0.41-3.12) | 0.81 |
| TVMI | 0.72% | 0.85% |  | 0.95 (0.12-7.46) | 0.96 |  | NA | NA |  | 0.47 (0.06-3.80) | 0.48 |

CDTLR= clinically driven target lesion revascularization; CDTVR = clinically driven target vessel revascularization; CI = confidence intervals; HR = hazard ratio; IPW = inverse probability weighted; IRA = infarct-related artery; NA = not applicable; TVF = target vessel failure; TVMI = target vessel related myocardial infarction.

*Based on robust sandwich variance estimates that cluster lesions within the same patients.

†Adjusted for covariates listed in Tables 1 and 2 as regressors of multivariable Cox and inverse probability weighted models.

**Supplementary Table 3: Adjusted Risk of Clinical Events Through 5 Years on a Patient Basis**

|  | **Cumulative 5-year incidence** | |  | **Crude HR** | |  | **Multivariable adjustment*** | |  | **IPW adjustment*** | |
| --- | --- | --- | --- | --- | --- | --- | --- | --- | --- | --- | --- |
| **Outcome** | **IRA** | **Non-IRA** |  | **HR (95% CI)** | ***p*** |  | **HR (95% CI)** | ***p*** |  | **HR (95% CI)** | ***p*** |
| TVF | 10.7% | 12.0% |  | 0.91 (0.51-1.60) | 0.72 |  | 1.30 (0.58-2.92) | 0.53 |  | 1.01 (0.43-2.40) | 0.98 |
| Cardiac death | 1.7% | 2.0% |  | 0.89 (0.21-3.81) | 0.87 |  | 1.77 (0.22-13.9) | 0.59 |  | 0.61 (0.08-4.34) | 0.62 |
| CDTVR | 9.3% | 10.4% |  | 0.90 (0.48-1.67) | 0.74 |  | 1.22 (0.50-3.02) | 0.66 |  | 1.12 (0.44-2.89) | 0.81 |
| TVMI | 0.78% | 0.97% |  | 0.89 (0.11-6.95) | 0.91 |  | NA | NA |  | 0.59 (0.07-4.78) | 0.62 |

CDTVR = clinically driven target vessel revascularization; CI = confidence intervals; HR = hazard ratio; IPW = inverse probability weighted; IRA = infarct-related artery; NA = not applicable; TVF = target vessel failure; TVMI = target vessel related myocardial infarction.

*Adjusted for covariates listed in Tables 1 and 2 as regressors of multivariable Cox and inverse probability weighted models.

**Supplementary Figure 1: Study Flowchart**


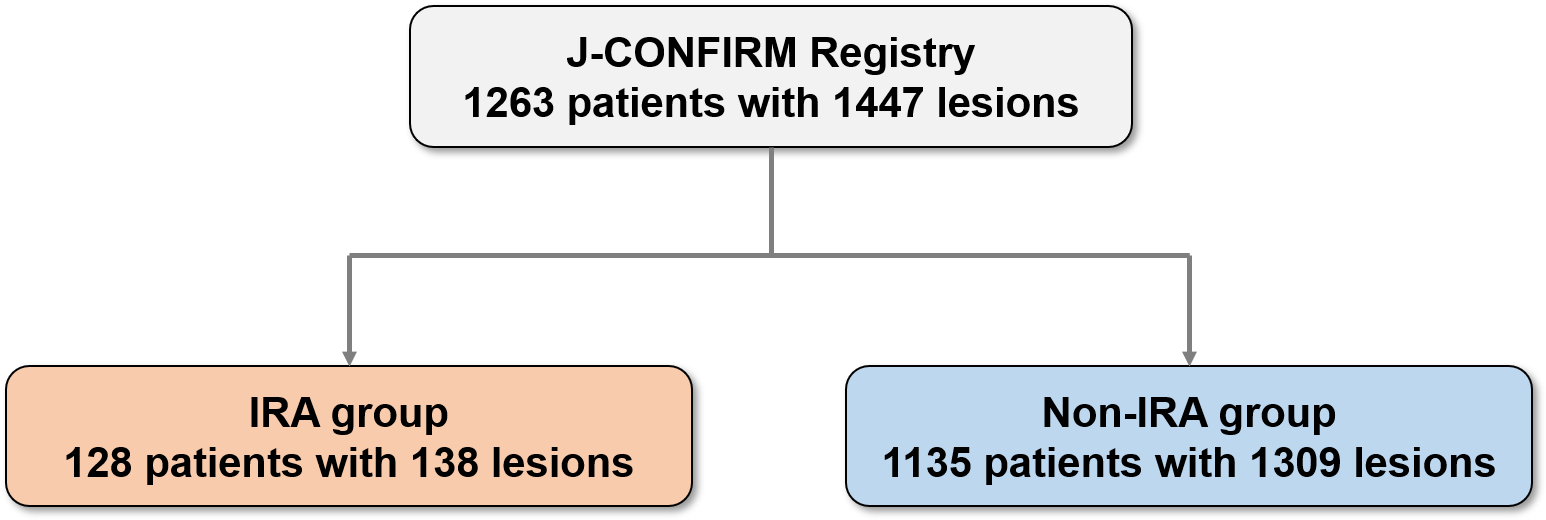


IRA = infarct-related artery

**Supplementary Figure 2:** **Comparison of FFR Values Between the IRA and Non-IRA Lesions According to Angiographic Diameter Stenosis**


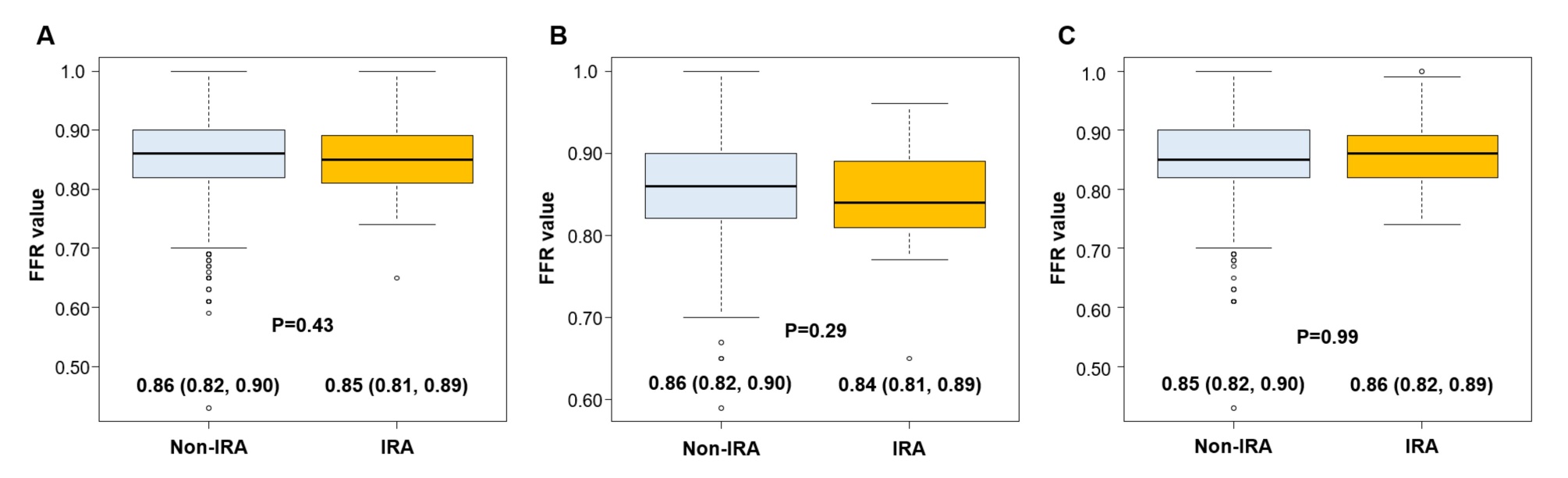


(A) overall lesions, (B) lesions with percent diameter stenosis (%DS) ≥50, and (C) lesions with %DS <50. Yellow and blue boxes indicate FFR values in the IRA and non-IRA groups. FFR = fractional flow reserve; IRA = infarct-related artery.

**Supplementary Figure 3: Clinical Events After Deferral of Revascularization Through 5 Years on a Patient Basis**


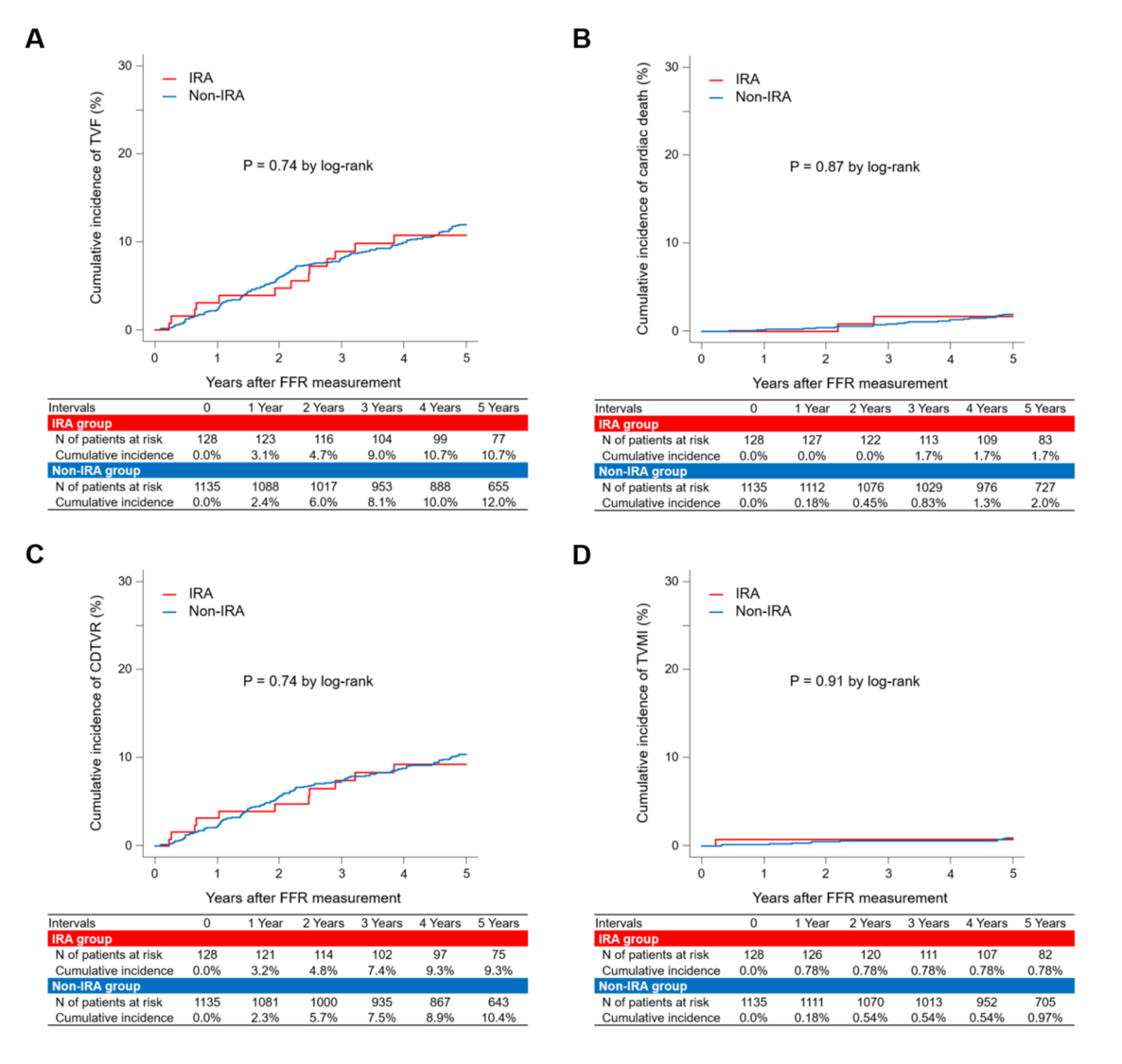


(A) target vessel failure (TVF), (B) cardiac death, (C) clinically driven target vessel revascularization (CDTVR), and (D) target vessel related myocardial infarction (TVMI). CI = confidence intervals; FFR = fractional flow reserve; IRA = infarct-related artery.

**Supplementary Figure 4: Correlation Between Angiographic Diameter Stenosis and FFR According to AMI Type**


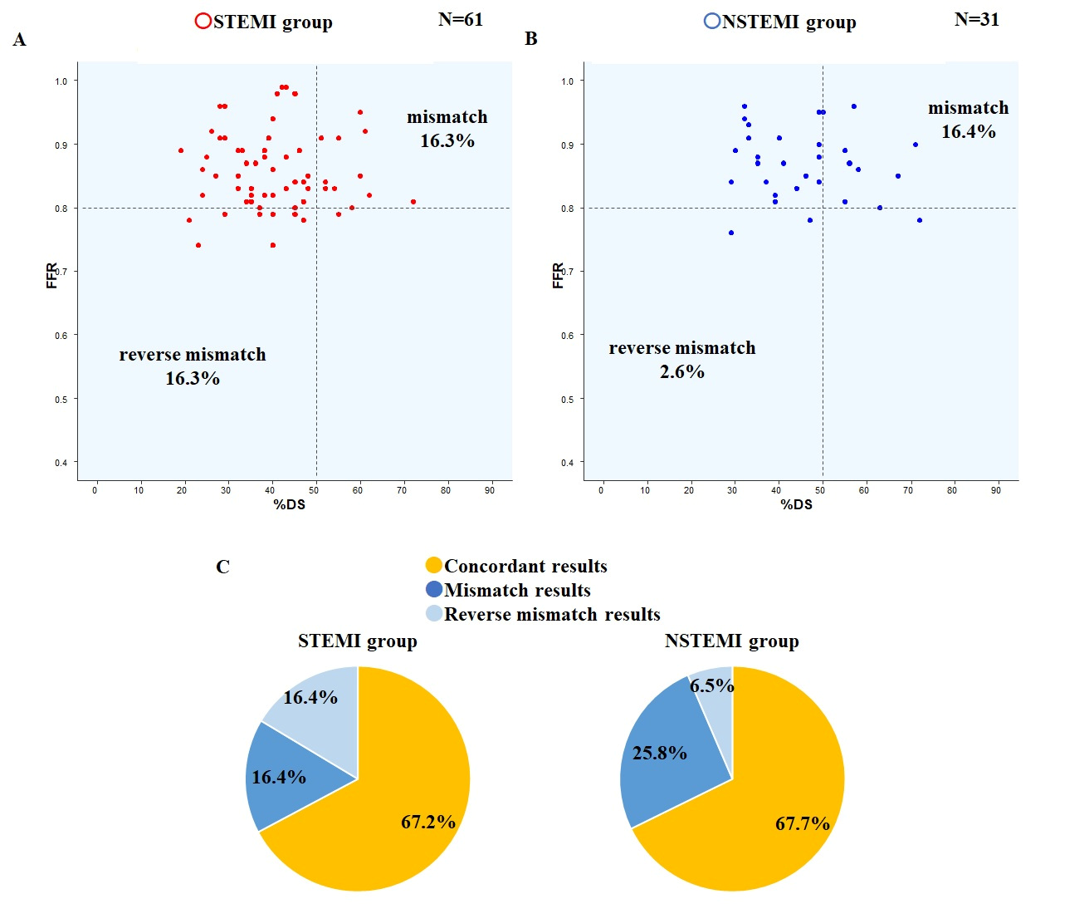


(A, B) correlation between percent diameter stenosis (%DS) and fractional flow reserve (FFR) in the STEMI and NSTEMI groups. (C) the frequency of discordance between angiographic diameter stenosis and FFR in both groups. Data on acute myocardial infarction (AMI) type or %DS were not available in 46 lesions. The difference and ratio between overall mismatch rates in the STEMI (20/61=32.8%) and NSTEMI (10/31=32.3%) groups were -0.5% (95% CI: -19.7% to 20.8%) and 1.02 (95% CI: 0.55 to 1.90), respectively.

NSTEMI = non-ST-segment myocardial infarction; STEMI = ST-segment elevation myocardial infarction.

**Supplementary Figure 5: Correlation Between Angiographic Diameter Stenosis and FFR According to Wall Motion Abnormalities**


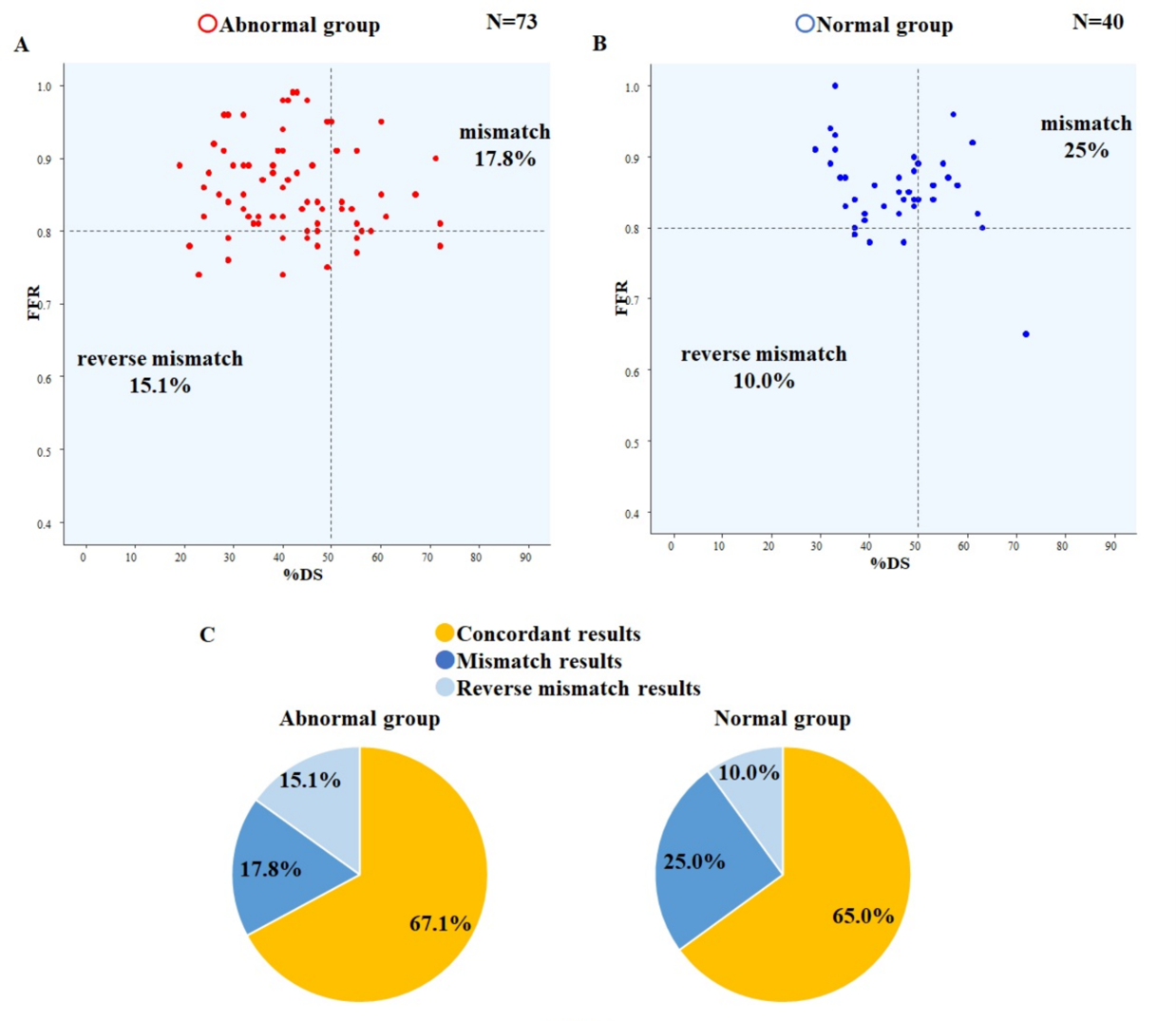


(A, B) correlation between percent diameter stenosis (%DS) and fractional flow reserve (FFR) in the abnormal and normal wall motion groups. (C) the frequency of discordance between angiographic diameter stenosis and FFR in both groups. Data on wall motion abnormalities or %DS were not available in 25 lesions. The difference and ratio between overall mismatch rates in the normal (24/73=32.9%) and abnormal (14/40=35.0%) groups were -2.1% (95% CI: -20.4% to 16.2%) and 0.94 (95% CI: 0.55 to 1.60), respectively.

**Supplementary Figure 6: Outcomes of Deferral of Revascularization in IRA Lesions According to Regional Wall Motion Abnormalities and Clinical Presentation**


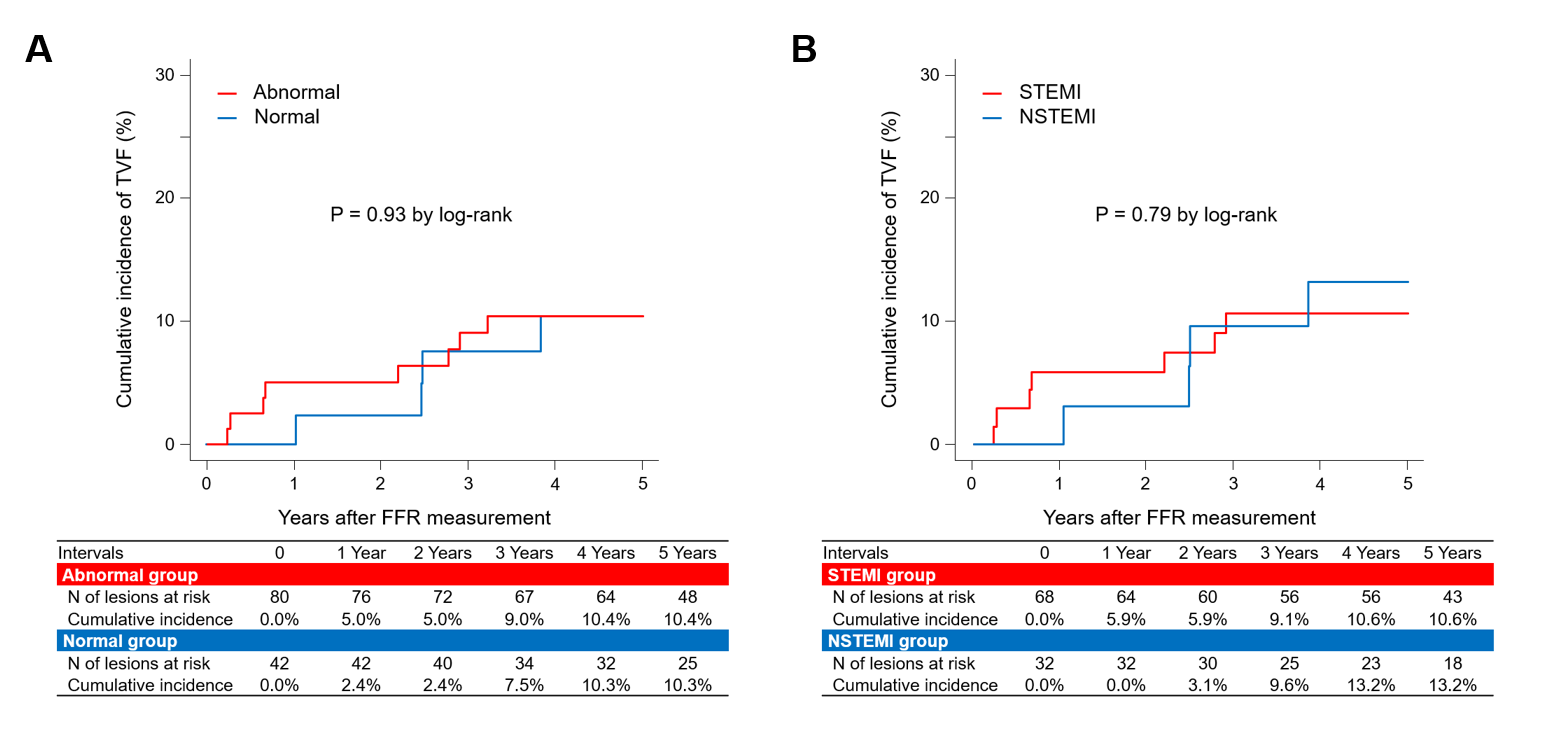


(A) regional wall motion abnormalities and (B) clinical presentation. Data on regional wall motion abnormalities and clinical presentation were not available in 16 and 38 lesions, respectively. FFR = fractional flow reserve; NSTEMI = non-ST-segment myocardial infarction; STEMI = ST-segment elevation myocardial infarction; TVF = target vessel failure.
